# A fully integrated sample-to-answer molecular diagnostic platform for rapid identification of four major *Aspergillus* species

**DOI:** 10.3389/fmicb.2026.1828196

**Published:** 2026-05-26

**Authors:** Eun Hae Oh, Jinseok Kang, Dasol Jin, Chaehwa Yoo, Jihoon Park, Eun Jeong Won

**Affiliations:** 1 iGENETECH R&D Center, iGENETECH Inc., Yongin, Republic of Korea; 2 Department of Laboratory Medicine, Asan Medical Center, University of Ulsan College of Medicine, Seoul, Republic of Korea; 3 Department of Molecular Genetics and Microbiology, Duke University Medical Center, Durham, NC, United States

**Keywords:** *Aspergillus* species identification, bronchial alveolar lavage, invasive pulmonary aspergillosis, MALDI-TOF, sample-to-answer platform

## Abstract

**Background:**

Invasive pulmonary aspergillosis (IPA) is more common among immunocompromised patients. Timely diagnosis is crucial but is challenging because current diagnostic approaches are often limited by low sensitivity, prolonged turnaround times, and technical complexity. Here, we developed a fully automated, real-time PCR–based, sample-to-answer molecular diagnostic platform for the simultaneous identification of four major *Aspergillus* species (*A. fumigatus, A. flavus, A. niger*, and *A. terreus*).

**Materials and methods:**

The sample-to-answer workflow was optimized using artificial bronchoalveolar lavage (BAL) samples spiked with *Aspergillus* species. The platform’s performance was evaluated through two primary validation steps: analytical consistency with MALDI-TOF using 21 patient-derived culture isolates, and clinical feasibility validation using 22 clinical BAL specimens from patients categorized as proven (*n* = 11) or probable (*n* = 11) IPA.

**Results:**

The assay demonstrated 95.2% (20/21) overall agreement with MALDI-TOF for culture isolates, achieving 93.3% positive agreement and 100% negative agreement. In the feasibility validation using 22 patient BAL samples including 11 proven and 11 probable IPA cases, the platform detected 10 out of 11 proven IPA samples (90.9%) with 100% species-level consistency with MALDI-TOF results. Notably, this assay could add mycological evidence in 3 of 11 culture-negative probable IPA cases.

**Conclusion:**

This fully automated, sample-to-answer platform enables rapid and accurate species-level identification of *Aspergillus* in suspicious IPA. We highlight that this assay offers a powerful molecular tool to overcome the limitations of conventional diagnostic methods in clinical settings.

## Introduction

Pulmonary aspergillosis (IPA) is a severe infection caused by *Aspergillus* species that invade the lungs or other organs, primarily affecting individuals with weakened immune systems, including patients with hematologic malignancies, recipients of hematopoietic stem cell or solid organ transplants, and those receiving immunosuppressive therapy (Husain and Camargo, 2019; Melenotte et al., 2023). Epidemiological studies indicate that IPA is among the most common invasive fungal infections in hospital settings after candidiasis and accounts for a substantial proportion of fungal-related morbidity and mortality. Recent estimates suggest that the global incidence of IPA exceeds 2 million cases annually, with approximately 1.8 million associated deaths (Mardani, 2024).

Early and accurate diagnosis is critical for improving clinical outcomes through timely administration of antifungal agents such as voriconazole or amphotericin B. However, conventional diagnostic methods, including culture and histopathological examination, are time-consuming and often lack sensitivity, particularly in patients who have already received antifungal therapy (Arvanitis et al., 2014; Patterson et al., 2016; Falci et al., 2017). Serological and antigen-based assays, such as galactomannan and β-D-glucan tests, provide more rapid results but are limited by cross-reactivity and variable sensitivity depending on the site of infection (Bretagne and Alanio, 2017; Hsu et al., 2021; Vanbiervliet et al., 2025). Matrix-assisted laser desorption/ionization time-of-flight mass spectrometry (MALDI-TOF MS) has become an important tool for fungal identification from cultured isolates, yet its accuracy depends heavily on completeness and quality of reference databases (Clark et al., 2013). Reliable species-level identification remains challenging for *Aspergillus* due to spectral similarity among closely related species, and fungal sample preparation for MALDI-TOF MS is labor-intensive and operator-dependent. Collectively, these limitations underscore the need for an alternative molecular diagnostic approach that enables simple, rapid, and accurate species identification directly from clinical specimens (White et al., 2015a; Kim et al., 2020).

To fill up this gap, we developed a user-friendly, fully integrated sample-to-answer molecular diagnostic platform for the identification of four major *Aspergillus* species, *A. fumigatus, A. flavus, A. niger, and A. terreus*, which together account for majority of IPA cases.

The system consists of an automated analyzer, MoiM Dx (iGENETECH Inc., Yongin, Korea), and a single-use cartridge preloaded with all reagents required for sample processing and target detection. This study aimed to introduce this novel molecular assay for diagnosis of IPA and address performance data of the assay using bronchoalveolar lavage samples.

## Materials and methods

### MoiM Dx platform

**FIGURE 1:**
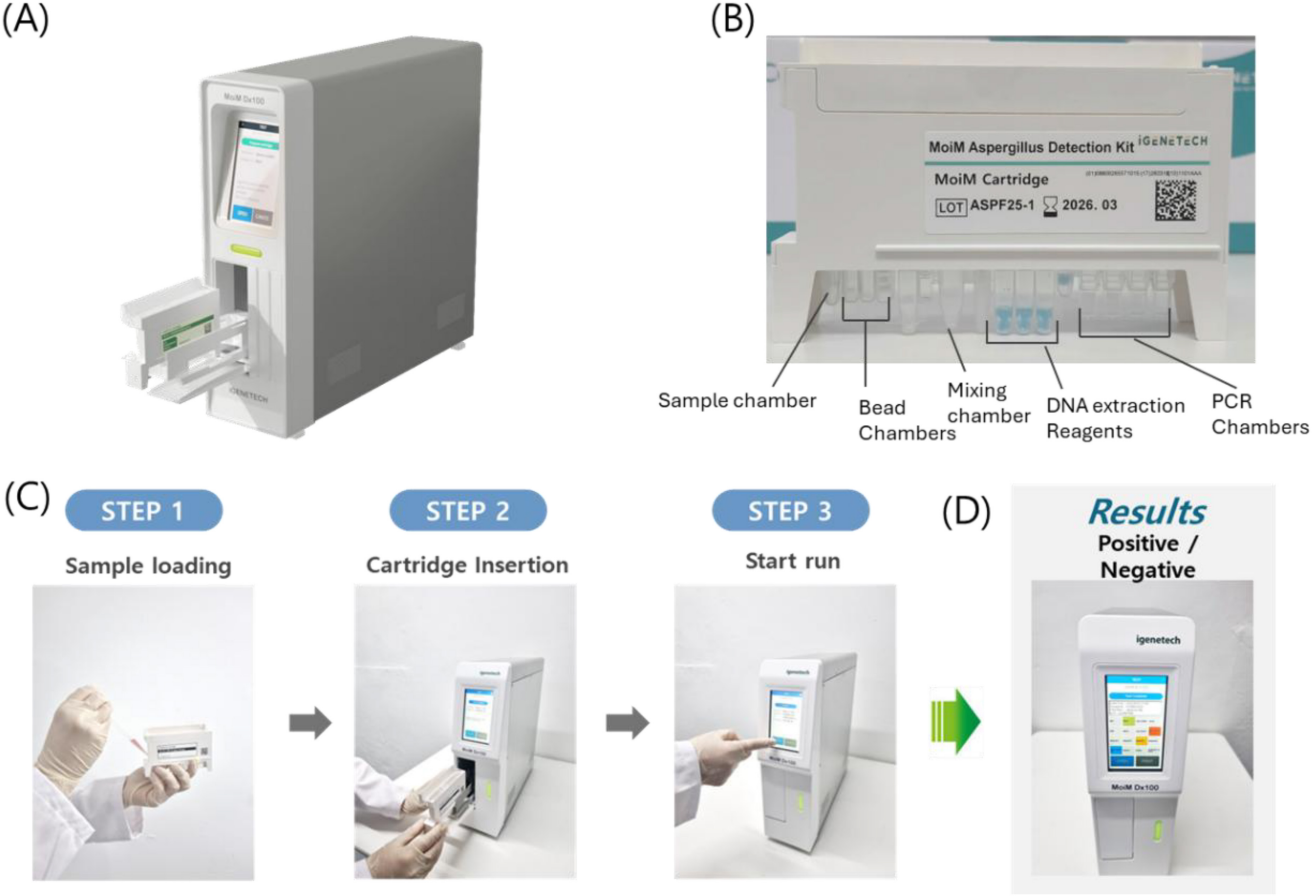
Overview of the MoiM Dx platform. **(A)** The MoiM Dx analyzer and disposable cartridge. **(B)** The cartridge containing all reagent required entire process from sample processing to PCR **(C)** sample-to-answer workflow; step 1, loading 200 μL of sample into the cartridge; step 2, cartridge insertion into the analyzer; step 3, initiation of automated run via touchscreen interface. **(D)** Result screen of the MoiM Dx platform displaying positive or negative outcomes upon completion of the integrated workflow.

The MoiM Dx platform (iGENETECH Inc., Yongin, Korea) consists of a fully automated analyzer and a single-use disposable cartridge (Figure 1A). The analyzer measures 104 mm (W) × 387.0 mm (D) × 360.5 mm (H), weighs 7.8 kg, and is equipped with a 4.3-inch touchscreen interface that enables users to control assay execution and monitor operational status and test results. The disposable cartridge contains all reagents required for sample processing, DNA extraction, target amplification, and fluorescence-based detection in a room temperature–stable format (Figure 1B). In the MoiM Dx platform, all liquid-handling operations are performed by an analyzer-driven pipetting mechanism, whereas the disposable cartridge serves exclusively as a reagent storage and reaction module. The cartridge measures 20 mm in width, 112 mm in length, and 76.5 mm in height, and incorporates a sample chamber, bead chambers, a mixing chamber, DNA extraction reagents, and four PCR chambers. Each PCR chamber is capable of detecting four different fluorescent signals, allowing for multiplex detection of up to 16 targets. In this study, a 5-plex assay was implemented, comprising four *Aspergillus* species and one internal process control. The sample-to-answer workflow is illustrated in Figure 1C. Briefly, 200 μL of sample is loaded into the cartridge (step 1), the cartridge is inserted into the analyzer (step 2), and the automated run is initiated via the touchscreen interface (step 3). Once initiated, all steps, including DNA extraction, PCR amplification, and signal detection, are performed automatically without further user intervention. Upon completion of the run, qualitative test results (positive or negative) are displayed on the analyzer screen (Figure 1D).

### Primer and probe design

Specific primers and hydrolysis probes were designed to target the *Cyp51A* gene of *Aspergillus fumigatus*, the *CaM* gene of *A. niger*, the *SCW4* gene of *A. flavus*, and the *CaM* gene of *A. terreus*. Complete gene sequences for each target were retrieved from the NCBI database and aligned using Jalview software to identify conserved regions suitable for primer and probe design. The designed primers and probes consisted of 17–35 nucleotides with a GC content of 40–70%, generating amplicons ranging from 75 to 200 base pairs in length. Since the MoiM Dx platform contains four independent PCR chambers, two multiplex assay groups were designed, with each group targeting two different *Aspergillus* species. Accordingly, different fluorophores were assigned to each probe: HEX for *A. fumigatus* and *A. niger*, and FAM for *A. flavus* and *A. terreus*. For the process control, *Saccharomyces cerevisiae* was selected, and specific primers and a probe labeled with Cy5 were designed and included in multiplex group 1. The specificity of each primer–probe set was evaluated *in silico* using the NCBI BLAST tool, confirming no significant cross-reactivity among the *Aspergillus* target genes.

### DNA extraction from fungal culture for primer/probe validation

To evaluate the amplification performance of each primer and probe set, lyophilized powders of four *Aspergillus* strains (*A. fumigatus, A. niger, A. flavus, and A. terreus*) were obtained from the Korean Collection for Type Cultures (KCTC, Korea). Each lyophilized sample was reconstituted in 3 mL of distilled water. Subsequently, 100 μL of the reconstituted suspension was added to 50 mL of YPD medium (Gibco, United States) and incubated overnight at 37°C with shaking. Genomic DNA was extracted from 1.5 mL of each fungal culture using the QIAmp UCP Pathogen Mini Kit (Qiagen, Germany) with Pathogen Lysis Tubes containing glass beads to ensure efficient fungal cell disruption. All genomic DNA extraction was performed in accordance with the manufacturer’s instructions.

### Confirmation of amplification performance of primers and probes

PCR amplification of each primer and probe set was performed using the Gentier96 real-time PCR system (Tianlong, China) with a 5x PCR premix (BioAssay, Korea). Genomic DNA extracted from each *Aspergillus* strain was serially diluted from 10*4* to 5 genome-equivalent copies per reaction. The thermal cycling conditions were as follows: initial denaturation at 95°C for 5 min, followed by 40 cycles of denaturation at 95°C for 2 s, annealing and extension at 57°C for 10 s. Amplification data were analyzed using the Gentier96 software, and the threshold cycle (Ct) values were automatically determined by the program based on baseline-subtracted curve fitting. The specificity of each primer and probe set was evaluated by testing the sets against genomic DNA from non-target *Aspergillus* species. After evaluating the amplification performance of each of the four *Aspergillus* targets individually in simpleplex format, the primer-probe sets were subsequently combined into two multiplex groups. Multiplex group 1 consisted of primer–probe sets targeting *A. fumigatus* and *A. flavus*, and multiplex group 2 consisted of primer–probe sets targeting *A. niger* and *A. terreus*. Multiplex amplification was performed under the same thermal cycling conditions as those used for the singleplex assays, and the resulting performance was compared with the corresponding singleplex reactions to confirm that multiplexing did not introduce assay interference or affect amplification performance. After confirming the amplification performance of each multiplex group, competitive amplification experiments were performed to evaluate potential competitive inhibition between target DNAs during multiplex amplification. Genomic DNA from each pair of target species was mixed at varying relative concentration ratios (10,000:1, 1,000:1, 100:1, 10:1, 1:1, 1:10, 1:100, 1:1,000, and 1:10,000), and multiplex PCR was performed using the MiC qPCR Cycler (Bio Molecular Systems, Australia) under the same thermal cycling conditions described above. Amplification curves and Ct values were analyzed to determine whether the presence of an excess amount of one target affected the amplification of the co-amplified target.

### Optimization of enzyme-mediated DNA extraction

Prior to implementing DNA extraction on the MoiM Dx platform, an enzyme-mediated lysis approach was evaluated to assess its suitability for integration into the sample-to-answer workflow. In-house-manufactured lyophilized lyticase beads (10 units per bead) and proteinase K beads (0.1 mg per bead) were used in combination with a commercial DNA extraction kit (BioPark Dx, South Korea). Genomic DNA extraction efficiency was evaluated using cultured *Saccharomyces cerevisiae*, which was employed as a process control organism due to its consistent growth and well-defined cell wall properties, enabling reproducible assessment of lysis and extraction performance. To assess enzyme-mediated DNA extraction efficiency from fungi, 200 μL aliquots of *S. cerevisiae* culture were prepared under three conditions: (i) addition of one lyticase bead and one proteinase K bead, (ii) addition of a proteinase K bead only, and (iii) no enzyme addition. Genomic DNA extraction efficiency was evaluated by quantitative PCR using *S. cerevisiae*-specific primers and probe, Ct values were compared across conditions. Those results were further compared with Ct value obtained from reference DNA extraction method, the QIAamp UCP Pathogen Mini Kit (Qiagen, United States) with Pathogen Lysis Tubes (Qiagen, United States).

### Sample-to-answer workflow optimization

To establish the sample-to-answer workflow on the MoiM platform, optimization of the PCR module was performed first, followed by integration of the DNA extraction process. Using the MoiM Dx developer software, thermal cycling parameters, including temperature and duration for pre-denaturation and denaturation, were adjusted, while annealing/extension conditions were kept constant (63°C for 20 s), to identify conditions compatible with fully automated operation. PCR premix for a single PCR chamber was prepared by solubilizing an in-house lyophilized PCR master bead with 10 μL of distilled water, followed by the addition of 5 μL of mixed genomic DNA preparation containing all four *Aspergillus* species as the template. Subsequently, two primer-probe sets corresponding to each multiplex group were added at 5 μL each. The prepared PCR premix was dispensed into individual PCR chambers of the cartridge and evaluated under various thermal cycling conditions. After determining the optimal PCR parameters, the DNA extraction protocol was integrated into the sample-to-answer workflow by applying the previously optimized enzyme-mediated DNA extraction conditions. To evaluate extraction performance within the fully automated system, cartridges prefilled with lyophilized enzyme beads (PCR master mix bead, lyticase bead, and proteinase K bead), a process control bead, magnetic beads, extraction reagents, and heat-dried primer–probe sets were used. Artificial bronchoalveolar lavage (BAL) samples (Biochemazone, Canada) spiked with a mixture of four *Aspergillus* species (*A. fumigatus, A. flavus, A. niger*, and *A. terreus*) were used as representative clinical matrices for workflow integration and performance evaluation. A total volume of 200 μL of each spiked artificial BAL sample was loaded into the sample chamber, and the integrated workflow was initiated using the MoiM Dx developer software. Parameters of the DNA extraction steps, including enzymatic lysis, washing, and elution, were further adjusted to achieve stable and reproducible DNA extraction and amplification performance across all targets. The conditions yielding the most consistent Ct values across all four *Aspergillus* species were selected as the final extraction protocol. For comparison, a conventional workflow was also performed using a standalone DNA extraction instrument (Tianlong, China) and a Gentier96 real-time PCR system (Tianlong, China), employing the same sample reagents used for the MoiM Dx platform to ensure concordance with the reference method.

### Specificity evaluation using non-target microorganisms

To assess analytical specificity, a panel of 20 non-target microbial species that were potentially encountered in respiratory specimens, clinical samples, or environmental sources were tested. The panel included *Streptococcus mutans*, *Escherichia coli*, *Staphylococcus aureus*, *Staphylococcus epidermidis*, *Staphylococcus warneri*, *Pseudomonas aeruginosa*, *Klebsiella pneumoniae*, *Alcaligenes faecalis* subsp. *faecalis*, *Proteus mirabilis*, *Bacillus cereus*, *Streptomyces griseoruber*, and multiple *Candida* species (*Candida albicans*, *Candida auris*, *Candida dubliniensis*, *Candida glabrata*, *Candida guilliermondii*, *Candida krusei*, *Candida parapsilosis*, *Candida tropicalis*, and *Candida lusitaniae*). Each strain was spiked into 200 μL of artificial BAL sample at a final concentration of 1 × 10^5^ genomic equivalent copies per reaction. The spiked samples were loaded into the MoiM Dx cartridge, and the fully automated sample-to-answer workflow was executed. Analytical specificity was assessed by monitoring the absence of amplification signals within 40 PCR cycles.

### Identification of *Aspergillus* isolates from clinical specimens

Representatively, a total of 21 clinical isolates of *Aspergillus* species including 5 isolates of *A. fumigatus*, 5 isolates of *A. flavus*, 4 isolates of *A. niger*, 3 isolates of *A. versicolor*, 2 isolates of *A. lentulus*, one isolate of *A. terreus*, and one isolate of *A. nidulans* were included in this study. All isolates were obtained from clinical specimens of patients from primary cultures on Sabouraud agar (Difco, Sabouraud Agar, Modified, BD, Sparks, MD, United States) during the study period (January 2024 to December 2024) at a tertiary medical center in South Korea containing 2,732 beds. Species identification was confirmed via matrix-assisted laser desorption/ionization time–of–flight mass spectrometry (MALDI-TOF MS; Bruker MALDI Sirius, Bruker Daltonics GmbH, Bremen, Germany) with the MBT Filamentous Fungi Library (version 4.0). Representative samples were prepared spiking colonies as follows: Colonies were collected from each culture plate and resuspended in 200 μL of PBS. The suspension was loaded into the sample chamber of the cartridge, and the fully automated sample-to-answer protocol was performed using the MoiM Dx 100 platform. Data from MoiM Dx was compared to those from MALDI-TOF, and then concordant or discordant results were determined. In case of non-target *Aspergillus* species, negative results by MoiM Dx were determined as categorically concordant data.

### Clinical BAL samples

Decisions to perform fiberoptic bronchoscopy with BAL were made by physicians. During the study period, fungal culture with BAL samples were routinely performed on samples from patients who underwent BAL with clinical suspicion of IPA. Remnant BAL samples were collected for further evaluation of the MoiM Dx 100 platform.

### Case definition and statistical analysis

Cases were classified as proven/probable IPA according to EORTC/MSG criteria being interpreted as having a positive result and cases with no evidence of IFD according to EORTC/MSG criteria being interpreted as having a negative result. Performance was evaluated to determine the negative predictive value (NPV), positive predictive value (PPV), sensitivity, and specificity for the true-positive and true-negative populations. Statistical analysis was performed using SPSS, version 20 (SPSS Inc., Chicago, IL, United States).

## Results

### Qualitative PCR analysis for confirmation of primer and probe performance

The target genes of each *Aspergillus* species were individually amplified using the corresponding primer–probe sets. Extracted genomic DNA was serially diluted and used as the PCR template. Consistent amplification across triplicate reactions was observed down to 10 genome-equivalent copies per reaction for *A. fumigatus* and 5 copies per reaction for *A. flavus*, *A. niger*, and *A. terreus* (Table 1). Although the cartridge contains four PCR chambers, two multiplex combinations were constructed to develop a cost-effective and efficient assay kit. Multiplex group 1 consisted of primer–probe sets for *A. fumigatus* and *A. flavus*, and multiplex group 2 included primer–probe sets for *A. niger* and *A. terreus*. To evaluate the multiplexing effect, the amplification performance of each target was compared with that of its corresponding singleplex PCR reaction. Ct values and the limit of detections (LoDs) obtained from the multiplex assays were equivalent to those from the singleplex assays (Table 1). No significant differences in Ct values were observed between the two formats, indicating that amplification efficiency was maintained under multiplex conditions. The CV% of Ct values remained below 5%, with slightly higher variability at LoD levels. Across the concentration range of 10^4^ to 10^1^ copies per reaction, CV% values were below 1% for all targets. The specificity of each primer–probe set was confirmed using both target and non-target *Aspergillus* species. As summarized in Supplementary Table S1, each primer–probe set amplified only its intended target species, and no cross-reactivity was observed among the *Aspergillus* species tested. To further evaluate potential amplification interference under multiplex conditions, both targets in each multiplex group were consistently detected across all tested relative concentration ratios (Supplementary Figure S1). Under assay conditions designed to detect the lowest target concentration with Ct values below 35, all tested target combinations were amplified without observable inhibition, even in the presence of excess co-amplified target DNA, indicating minimal target-to-target interference during multiplex amplification.

**TABLE 1:** Amplification performance of singleplex and multiplex PCR assays for four *Aspergillus* targets.

Copies /reaction	Multiplex group 1 (*A. fumigatus* and *A. flavus*)						Copies/ reaction	Multiplex group 2 (*A. niger and A. terreus)*					
	*A. fumigatus* (Mean Ct)			*A. flavus* (Mean Ct)				*A. niger* (Mean Ct)			*A. terreus* (Mean Ct)		
	Singleplex	Multiplex	CV (%)	Singleplex	Multiplex	CV (%)		Singleplex	Multiplex	CV (%)	Singleplex	Multiplex	CV (%)
10^4^	26.11 (3/3)	25.99 (3/3)	0.35	24.61 (3/3)	24.39 (3/3)	0.65	10^4^	24.89 (3/3)	24.61 (3/3)	0.80	24.11 (3/3)	23.98 (3/3)	0.38
10^3^	30.51 (3/3)	30.34 (3/3)	0.39	28.04 (3/3)	27.96 (3/3)	0.18	10^3^	28.21 (3/3)	28.09 (3/3)	0.32	27.46 (3/3)	27.31 (3/3)	0.39
10^2^	34.23 (3/3)	33.94 (3/3)	0.61	30.65 (3/3)	31.14 (3/3)	1.12	10^2^	31.49 (3/3)	31.31 (3/3)	0.40	30.78 (3/3)	30.76 (3/3)	0.05
10^1^	36.03 (3/3)	36.18 (3/3)	0.31	34.37 (3/3)	34.48 (3/3)	0.24	10^1^	35.04 (3/3)	34.82 (3/3)	0.45	34.43 (3/3)	34.47 (3/3)	0.09
5	-	-	-	35.99 (3/3)	34.42 (3/3)	3.15	5	35.37 (3/3)	37.76 (3/3)	4.62	34.60 (3/3)	34.97 (3/3)	0.75
DW	-	-	-	-	-	-	DW	-	-	-	-	-	-

Ct, cycle threshold; CV, coefficient of variation; DW, distilled water; SD, standard deviation.

### Optimization of enzyme-based DNA extraction for integration into the MoiM Dx platform

Enzyme-mediated DNA extraction efficiency was evaluated using *Saccharomyces cerevisiae* as a fungal process control organism (Table 2). Among the tested conditions, combined treatment with lyticase and proteinase K resulted in the lowest mean Ct value (27.86 ± 0.56), indicating the highest DNA extraction efficiency. Treatment with proteinase K alone yielded higher Ct value (30.99 ± 1.05), while samples processed without enzymatic pretreatment showed the poorest performance, with the highest Ct values (34.95 ± 0.54). The enzyme-mediated condition using both lyticase and proteinase K produced Ct values comparable to those obtained using the reference DNA extraction method, the QIAamp UCP Pathogen Mini Kit with Pathogen Lysis Tubes (Ct = 28.19). No amplification was observed in the no-template control (NTC), confirming the absence of contamination. Overall, the enzyme-mediated lysis approach using lyophilized lyticase and proteinase K beads achieved DNA extraction efficiency comparable to a commercial reference kit, supporting its suitability for integration into the MoiM Dx sample-to-answer workflow.

**TABLE 2:** Evaluation of DNA extraction efficiency using *S. cerevisiae* as an internal process control.

Condition		Ct value		
		Replicate 1	Replicate 2	Mean Ct ± SD
Enzyme-mediated	Lyticase + proteinase K	28.25	27.46	27.86 ± 0.56
	Proteinase K only	30.24	31.73	30.99 ± 1.05
	Without enzymes	34.56	35.33	34.95 ± 0.54
Qiagen UCP pathogen kit with lysis beads		28.19	-	-
NTC		ND	ND	-

Ct, cycle threshold; SD, standard deviation; NTC, no template control; ND, not detected.

### Integration and optimization of the sample-to-answer workflow

To establish the complete sample-to-answer workflow, the PCR conditions of the MoiM Dx platform were first optimized using mixed genomic DNA from the four *Aspergillus* species at low concentrations. The temperatures and durations of the pre-denaturation and denaturation steps were adjusted through the MoiM Dx developer software, and the optimal PCR protocol was determined to be pre-denaturation at 95°C for 5 min, denaturation at 95°C for 9 s, and annealing/extension at 63°C for 20 s (see Supplementary Table S2). Following PCR optimization, the DNA extraction protocol was integrated into the workflow using a cartridge pre-filled with lyophilized reagents (PCR master mix bead, lyticase bead, proteinase K bead, process control bead and magnetic bead), liquid extraction buffers, and heat-dried primer–probe sets. The fully integrated MoiM Dx workflow was then evaluated using artificial BAL samples spiked with all four *Aspergillus* species. During integration into the sample-to-answer workflow, the extraction conditions were further adjusted; however, the overall enzymatic lysis strategy was maintained. When compared with the reference extraction followed by conventional real-time PCR, the MoiM Dx workflow produced similar Ct values for *A. fumigatus* and *A. terreus* (difference < 1 cycle), whereas *A. flavus* and *A. niger* exhibited Ct delays of approximately 3–4 cycles, indicating species-dependent variation in extraction efficiency. Despite this shift, all four targets as well as the internal process control were consistently detected within 40 cycles with low variability, confirming successful integration of extraction and amplification within the cartridge (Table 3). This evaluation was performed at a single representative concentration to verify functional workflow integration rather than to assess analytical sensitivity. The total turnaround time for sample-to-answer workflow was approximately 120 min.

**TABLE 3:** Performance evaluation of the MoiM Dx workflow compared with the reference method.

Method	Target detection (Ct value ± SD)				Process control
	*A. fumigatus*	*A. flavus*	*A. niger*	*A. terreus*	*S. cerevisiae*
Reference ^a^ (mean ± SD)	31.98 ± 0.31	32.60 ± 0.33	34.98 ± 0.07	35.06 ± 0.56	29.32 ± 0.72
MoiM Dx (mean ± SD)	31.02 ± 0.12	36.43 ± 0.01	39.20 ± 1.06	35.51 ± 0.07	29.02 ± 0.34

^a^Conventional workflow was used for the reference method, using a standalone DNA extraction instrument (Tianlong, China) and a Gentier96 real-time PCR system (Tianlong, China), employing the same sample reagents used for the MoiM Dx platform. Ct, cycle threshold; SD, standard deviation.

### Evaluation of assay specificity using non-target microorganisms

Analytical specificity was evaluated using a panel of 20 non-target microbial species spiked into artificial BAL samples at a concentration of 1 × 10^5^ genomic equivalent copies per reaction. No amplification signals were observed for any non-target microorganisms within 40 PCR cycles, indicating the absence of cross-reactivity under the tested conditions (Supplementary Table S3).

### Identification of patient-derived *Aspergillus* culture isolates

The detection and identification performance of the MoiM Dx *Aspergillus* 4-plex assay was evaluated using *Aspergillus* cultures isolated from patient BAL specimens and compared with MALDI-TOF results. A total of 21 culture isolates were tested. Based on MALDI-TOF identification, five cultures were identified as *A. fumigatus*, five as *A. flavus*, four as *A. niger*, one as *A. terreus*, and the remaining six as non-target *Aspergillus* species (*A. lentulus*, *A. versicolor*, and *A. nidulans*) (Table 4). Using the MoiM Dx platform, 14 of the 15 target isolates were correctly identified, with one *A. fumigatus* isolate yielding a negative result. All non-target *Aspergillus* species were reported as negative, and designated into categorically concordant. This corresponded to an overall agreement of 95.2% (20/21), with a positive percent agreement of 93.3% (14/15) for target species and a negative percent agreement of 100% (6/6) for non-target species. In all reactions, the internal process control included in the MoiM Dx assay was successfully detected, confirming valid assay performance.

**TABLE 4:** Identification results of MoiM *Aspergillus* 4-plex and of 21 cultured *Aspergillus* isolates.

No	Identification results		Concordance^a^
	MALDI-TOF	MoiM *Aspergillus* 4-plex	
1	*A. fumigatus*	*A. fumigatus*	Concordant
2	*A. fumigatus*	*A. fumigatus*	Concordant
3	*A. fumigatus*	*A. fumigatus*	Concordant
4	*A. fumigatus*	*A. fumigatus*	Concordant
5	*A. fumigatus*	Negative	Discordant
6	*A. flavus*	*A. flavus*	Concordant
7	*A. flavus*	*A. flavus*	Concordant
8	*A. flavus*	*A. flavus*	Concordant
9	*A. flavus*	*A. flavus*	Concordant
10	*A. flavus*	*A. flavus*	Concordant
11	*A. niger*	*A. niger*	Concordant
12	*A. niger*	*A. niger*	Concordant
13	*A. niger*	*A. niger*	Concordant
14	*A. niger*	*A. niger*	Concordant
15	*A. terreus*	*A. terreus*	Concordant
16	*A. lentulus*	Negative (non-target species)	Categorically concordant
17	*A. lentulus*	Negative (non-target species)	Categorically concordant
18	*A. versicolor*	Negative (non-target species)	Categorically concordant
19	*A. versicolor*	Negative (non-target species)	Categorically concordant
20	*A. versicolor*	Negative (non-target species)	Categorically concordant
21	*A. nidulans*	Negative (non-target species)	Categorically concordant

^a^Data from MoiM Dx was compared to those from MALDI-TOF, and then concordant or discordant results were determined. In case of non-target *Aspergillus* species, negative results by MoiM Dx were determined as categorically concordant data.

### Feasibility evaluation using patient BAL samples

A total of 22 BAL samples were tested directly using the MoiM assay. Of these, 11 samples exhibited high galactomannan (GM) optical density values ( > 10.0) and were culture-positive for *Aspergillus* species; these were classified as proven IPA. The remaining 11 samples showed elevated or borderline GM values (0.63 to > 10.0) but were culture-negative and were categorized as probable IPA based on compatible clinical findings and receipt of antifungal therapy (Table 5 and Supplementary Table S4). For analysis using the MoiM assay, a Ct value below 40 cycles was used to define positive detection. Direct MoiM testing of the BAL samples presented positive results in 10 of 11 proven IPA cases, and 3 of 11 probable IPA cases (two *A. fumigatus* and one *A. flavus*), respectively. One proven IPA sample identified as *A. fumigatus* by culture was not detected by the MoiM assay. To further assess assay specificity in non-infected individuals, an additional 67 BAL samples from patients without antifungal therapy or clinical signs of IPA were tested. Among these samples, three were positive by the MoiM *Aspergillus* 4-plex assay (two *A. flavus* and one *A. terreus*), all of which were culture-negative (Supplementary Table S5).

**TABLE 5:** Summary of MoiM *Aspergillus* 4-plex results in BAL samples obtained from invasive pulmonary aspergillosis (IPA) patients.

Category	No. of cases	Galactomannan test (O.D. results)	Culture results	MoiM Aspergillus 4-plex	Concordance
Proven IPA	7	>10.0	*A. fumigatus*	*A. fumigatus*	Concordant
	1	>10.0	*A. fumigatus*	*A. fumigatus/A. flavus*	Concordant
	1	>10.0	*A. fumigatus*	Negative	Discordant, false negative
	1	>10.0	*A. fumigatus*	*A. fumigatus/A. terreus*	Concordant
	1	>10.0	*A. niger*	*A. niger*	Concordant
Probable IPA	2	>10.0	Nogrowth	Negative	Concordant
	1	3.40	No growth	Negative	Concordant
	1	1.06	No growth	Negative	Concordant
	1	1.01	No growth	Negative	Concordant
	1	0.63	No growth	Negative	Concordant
	1	>10.0	Yeast, not *Cryptococcus*	Negative	Concordant
	1	1.29	Yeast, not *Cryptococcus*	Negative	Concordant
	1	>10.0	Nogrowth	*A. fumigatus*	Discordant^a^
	1	1.76	No growth	*A. flavus*	Discordant^a^
	1	0.69	No growth	*A. fumigatus*	Discordant^a^

^a^The three cases of discordancy indicate possibility of additional mycological evidence by MoiM assay, than culture results.

## Discussion

The fungal culture, microscopy, and histopathology remain the gold standard methods for identification of the pathogenic fungi, gathering mycological evidence to obtain a definitive *proven* diagnosis (Mendonça et al., 2022). Culture can provide a proven diagnosis by demonstrating the presence of the live fungi in specimens, and it allows for the precise identification and antifungal susceptibility testing of the causative pathogen. Many routine laboratories utilize fungal culture followed by MALDI-TOF analysis, but it still takes several days to produce the results. Consequently, species-level identification is recognized as less useful for the timely routine practice, as MALDI-TOF requires viable colonies and involves multiple manual steps after culture, including colony selection, organic solvent–based protein extraction, matrix application, and spectrum interpretation. These labor-intensive procedures increase hands-on time and introduce variability related to extraction quality and database coverage. As a result, most patients are diagnosed and treated without species-level identification, despite the clinical relevance of species-specific antifungal susceptibility patterns and the importance of epidemiologic surveillance. These gaps have encouraged the development of molecular diagnostics capable of directly detecting *Aspergillus* nucleic acids and distinguishing clinically relevant species.

*Aspergillus* polymerase chain reaction testing of blood and BAL has been recently accepted as a mycological criterion for probable invasive aspergillosis in the second revision of the EORTC/MSGERC definitions for classifying invasive fungal disease (Donnelly et al., 2020). This inclusion is based on the significant progress that has been made in the standardization of *Aspergillus* PCR methodology, the availability of commercial assays, and increased confidence in performance as highlighted by a Cochrane review (Cruciani et al., 2019). A variety of research-use-only and/or CE-marked commercial kits for the direct detection of *Aspergillus* nucleic acid have been described (White et al., 2015b). However, any molecular assays for *Aspergillus* have not been approved yet by the U.S. Food and Drug Administration (FDA) for use in the diagnosis of IA. Molecular testing in the United States has, therefore, relied on laboratory-developed tests (LDTs), which also vary widely in terms of test formats and performance (Jenks et al., 2023). For clarification of the assay, considerable standardization should focus on the rate-limiting extraction protocol, qPCR targeting the multi-copy ribosomal DNA region (e.g., 18S, Internal transcribed spacers, 28S), duplicate testing of DNA eluates, and inclusion of an internal control to monitor for inhibition improve performance. This newly developed assay was operated in the cartridge type automated analyzer, integrating enzymatic lysis, magnetic bead–based nucleic acid extraction, quantitative PCR amplification, and fluorescence-based detection. This also enables minimal hands-on time with a more compact, maintenance-free device architecture and overall ease of use while reducing operator-related variability. Moreover, the system consists of an analyzer equipped with a pipette-based liquid handling module and a cartridge that serves solely as a reagent reservoir and reaction chamber. Due to this streamlined architecture which lacks complicated internal fluidic structures, the system can offer significantly lower testing and maintenance costs, thereby enhancing its practical applicability in clinical settings. Despite these advantages, certain analytical limitations were observed during system evaluation. The sample-to-answer evaluation demonstrated that DNA-recovery efficiency varied by target species within the on-cartridge extraction process. Such species-dependent differences in extraction efficiency may influence sensitivity in clinical samples with low fungal burden. Further evaluation across a broader range of fungal loads, particularly near the detection limit, would provide additional insight into species-dependent extraction efficiency and its impact on analytical sensitivity, thereby guiding further optimization of the extraction module to enhance overall assay performance, particularly for low-concentration specimens. In addition, the choice of molecular targets may also influence assay sensitivity and specificity. While multicopy targets, such as ribosomal DNA regions, may provide enhanced analytical sensitivity, they often lack sufficient resolution for reliable species-level discrimination. In this study, low-copy targets (*Cyp51A*, *CaM*, and *SCW4*) were deliberately selected to enable accurate identification of clinically relevant *Aspergillus* species. This design represents a trade-off between sensitivity and specificity and may contribute to reduced detection sensitivity in samples with low fungal burden.

The MoiM Dx *Aspergillus* 4-plex assay showed a good accuracy (95.2%) to MALDI-TOF results using artificial BAL samples. Importantly, when applied to clinical BAL specimens, the assay successfully detected target species in proven IA cases. Furthermore, among BAL samples from patients who exhibited clinical symptoms of infection and had already received antimicrobial therapy, the assay provided species-level identification in 27.3% (3/11) of culture-negative specimens. This capability highlights the potential clinical value of the assay in situations where conventional culture and MALDI-TOF fail to yield an organism. However, in culture-negative cases, the detection of *Aspergillus* DNA should be interpreted with caution, as positive results may reflect colonization rather than true invasive infection. In particular, differentiation between colonization and infection cannot be reliably achieved based solely on Ct values. Therefore, assay results should be interpreted in conjunction with clinical findings.

Taken together, these findings suggest that the MoiM *Aspergillus* 4-plex assay could serve as a practical and informative diagnostic tool for both culture-positive and culture-negative cases of suspected aspergillosis. Direct MoiM testing of the BAL samples detected 10 of 11 proven IPA cases, corresponding to a detection rate of 90.9%, and all detected cases showed species-level concordance with culture/MALDI-TOF identification. Two BAL samples produced dual-target results (e.g., *A. fumigatus/A. flavus* and *A. fumigatus/A. terreus*), indicating mixed *Aspergillus* colonization or co-infection in the airways. It is noteworthy that two *A. fumigatus* and one *A. flavus* were found in three cases of probable IPA, but no growth in previous culture. These suggest that the MoiM assay could give additional mycological evidence in such cases that are GM positive but culture negative, where culture often fails because of low fungal burden or prior antifungal treatment. We should be cautious in the interpretation due to possibility of false positive in case of airway colonization, transient environmental exposure, or the presence of non-viable fungal DNA rather than true invasive disease.

This study has several limitations. First, the study was designed to develop a detection platform for the major *Aspergillus* species, and the analysis of clinical BAL samples was performed primarily to explore potential applicability. As a result, the number of clinical specimens was limited and was not intended to support full clinical validation. Second, although the assay performed well in culture-positive isolates, its behavior in samples with very low fungal burden requires further characterization. The occasional detection of *Aspergillus* DNA in asymptomatic, culture-negative individuals suggests that background airway colonization or environmental exposure may influence results, and this warrants more detailed investigation. Third, because the platform relies on direct molecular detection, further refinement of sample processing for low-burden specimens may be needed, including improved lysis efficiency, selective enrichment of fungal elements, or methods to increase nucleic acid concentration. Finally, the absence of longitudinal clinical follow-up limits the ability to determine the clinical relevance of low-level detections. Larger multicenter studies will be required to define appropriate clinical use cases and to establish clearer interpretive criteria. With further optimization and clinical validation, this platform is expected to support timely and appropriate clinical decision-making by enabling species-level identification of *Aspergillus* directly from respiratory specimens.

## Data Availability

The original contributions presented in the study are included in the article/Supplementary material, further inquiries can be directed to the corresponding author.

## References

[B1] Arvanitis M. Anagnostou T. Fuchs B. B. Caliendo A. M. Mylonakis E. (2014). Molecular and nonmolecular diagnostic methods for invasive fungal infections. *Clin. Microbiol. Rev.* 27 490–526. 10.1128/CMR.00091-13 24982319 PMC4135902

[B2] Bretagne S. Alanio A. (2017). Challenges in microbiological diagnosis of invasive *Aspergillus* infections. *F1000Res.* 6:F1000 Faculty Rev-157. 10.12688/f1000research.10216.1 28299183 PMC5321116

[B3] Clark A. E. Kaleta E. J. Arora A. Wolk D. M. (2013). Matrix-assisted laser desorption ionization-time of flight mass spectrometry: A fundamental shift in the routine practice of clinical microbiology. *Clin. Microbiol. Rev.* 26 547–603. 10.1128/CMR.00072-12 23824373 PMC3719498

[B4] Cruciani M. Mengoli C. Barnes R. Donnelly J. P. Loeffler J. Jones B. L. et al. (2019). Polymerase chain reaction blood tests for the diagnosis of invasive aspergillosis in immunocompromised people. *Cochrane Database Syst. Rev.* 9:CD009551. 10.1002/14651858.cd009551.pub4 26343815

[B5] Donnelly J. P. Chen S. C. Kauffman C. A. Steinbach W. J. Baddley J. W. Verweij P. E. et al. (2020). Revision and update of the consensus definitions of invasive fungal disease from the European Organization for Research and Treatment of Cancer and the Mycoses Study Group Education and Research Consortium. *Clin. Infect. Dis.* 71 1367–1376. 10.1093/cid/ciz1008 31802125 PMC7486838

[B6] Falci D. R. Stadnik C. M. B. Pasqualotto A. C. (2017). A review of diagnostic methods for invasive fungal diseases: Challenges and perspectives. *Infect. Dis. Ther.* 6 213–223. 10.1007/s40121-017-0154-1 28357708 PMC5446367

[B7] Hsu A. J. Tamma P. D. Zhang S. X. (2021). Challenges with utilizing the 1,3-beta-D-glucan and galactomannan assays to diagnose invasive mold infections in immunocompromised children. *J. Clin. Microbiol.* 59:e0327620. 10.1128/JCM.03276-20 33883182 PMC8373034

[B8] Husain S. Camargo J. F. (2019). Invasive aspergillosis in solid-organ transplant recipients: Guidelines from the American Society of Transplantation infectious diseases community of practice. *Clin. Transplant.* 33:e13544. 10.1111/ctr.13544 30900296

[B9] Jenks J. D. White P. L. Kidd S. E. Goshia T. Fraley S. I. Hoenigl M. et al. (2023). An update on current and novel molecular diagnostics for the diagnosis of invasive fungal infections. *Expert Rev. Mol. Diagn.* 23 1135–1152. 10.1080/14737159.2023.2267977 37801397 PMC10842420

[B10] Kim W. B. Park C. Chun H. S. Lee D. G. (2020). Development of multiplex real-time PCR for rapid identification and quantitative analysis of *Aspergillus* species. *PLoS One* 15:e0229561. 10.1371/journal.pone.0229561 32150555 PMC7062252

[B11] Mardani M. (2024). Change in global incidence and mortality of invasive fungal infection. *Arch. Clin. Infect. Dis.* 19:e146136. 10.5812/archcid-146136

[B12] Melenotte C. Aimanianda V. Slavin M. Aguado J. M. Armstrong-James D. Chen Y. C. et al. (2023). Invasive aspergillosis in liver transplant recipients. *Transpl. Infect. Dis.* 25:e14049. 10.1111/tid.14049 36929539

[B13] Mendonça A. Santos H. Franco-Duarte R. Sampaio P. (2022). Fungal infections diagnosis – Past, present and future. *Res. Microbiol.* 173:103915. 10.1016/j.resmic.2021.103915 34863883 PMC8634697

[B14] Patterson T. F. Thompson G. R. Denning D. W. Fishman J. A. Hadley S. Herbrecht R. et al. (2016). Practice guidelines for the diagnosis and management of aspergillosis: 2016 update by the Infectious Diseases Society of America. *Clin. Infect. Dis.* 63 e1–e60. 10.1093/cid/ciw326 27365388 PMC4967602

[B15] Vanbiervliet Y. Aerts R. Maessen L. Wauters J. Maertens J. Lagrou K. (2025). Laboratory innovations to diagnose invasive mould infections—what is relevant, what is not? *Clin. Microbiol. Infect.* 32 715–728. 10.1016/j.cmi.2025.10.017 41173342

[B16] White P. L. Barnes R. A. Springer J. Klingspor L. Cuenca-Estrella M. Morton C. O. et al. (2015a). Clinical performance of *Aspergillus* PCR for testing serum and plasma: A study by the European *Aspergillus* PCR Initiative. *J. Clin. Microbiol.* 53 2832–2837. 10.1128/JCM.00905-15 26085618 PMC4540904

[B17] White P. L. Wingard J. R. Bretagne S. Löffler J. Patterson T. F. Slavin M. A. et al. (2015b). *Aspergillus* polymerase chain reaction: Systematic review of evidence for clinical use in comparison with antigen testing. *Clin. Infect. Dis.* 61 1293–1303. 10.1093/cid/civ507 26113653 PMC4583581

