# Supplementary material for: A fully integrated sample-to-answer molecular diagnostic platform for rapid identification of four major Aspergillus species
Source: Front Microbiol. 2026 May 26;17:1828196. doi: 10.3389/fmicb.2026.1828196 (PMC13246479; doi:10.3389/fmicb.2026.1828196)
Supplement: Supplementary file 1 [file Table_1.pdf]

Supplementary Figure S1. Competitive amplification of fungal DNA under varying relative input ratios.

(A) Multiplex group 1 (*A. fumigatus* and *A. flavus*)

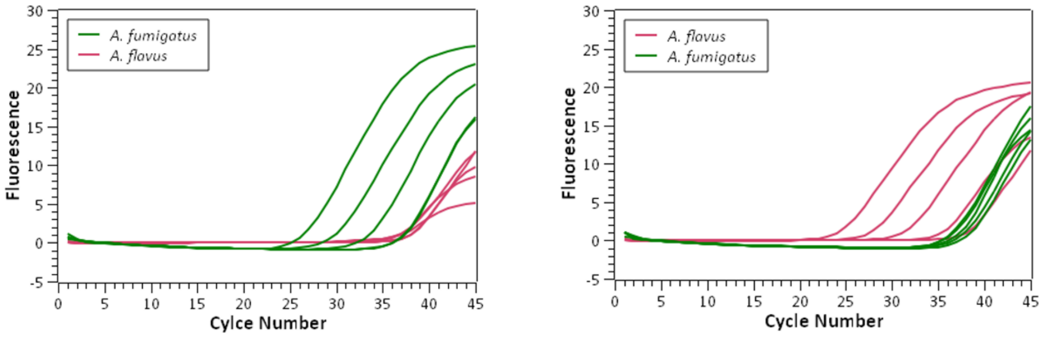

Relative input ratio (*A.fumigatus*: *A. flavus*)	Ct value (*A.fumigatus*)	Ct value (*A. flavus*)
10,000:1	26.07	35.34
1,000:1	29.57	35.12
100:1	33.22	35.48
10:1	36.98	36.81
1:1	37.06	36.88
1:10	37.98	34.10
1:100	36.01	29.62
1:1,000	37.29	26.18
1:10,000	36.31	22.71

(B) Multiplex group 2 (*A. niger* and *A. terreus*)

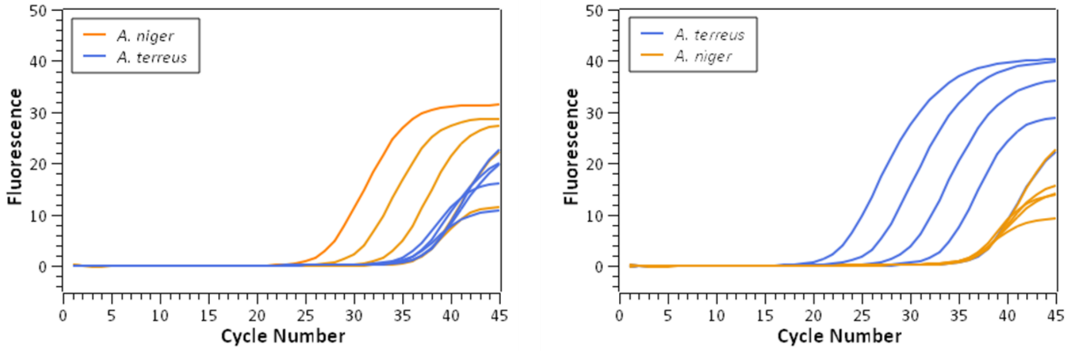

Relative input ratio (*A. niger* : *A. terreus*)	Ct value (*A. niger*)	Ct value (*A. terreus*)
10,000:1	25.15	36.49
1,000:1	28.47	35.56
100:1	32.18	37.19
10:1	35.80	36.39
1:1	35.97	37.28
1:10	35.22	32.60
1:100	35.56	29.05
1:1,000	35.53	25.34
1:10,000	35.17	21.90

Amplification curves and corresponding mean Ct values obtained from competitive multiplex PCR assays using

varying relative template ratios of target species. Input ratios represent relative template concentrations

Supplementary Table S1. Cross-reactivity evaluation of four individual *Aspergillus* primer–probe sets

Strain	Primer-Probe Set (Ct value)			
	A. fumigatus	*A. niger*	*A. flavus*	*A. terreus*
A. fumigatus	30.11	–	–	–
A. niger	–	28.09	–	–
A. flavus	–	–	27.61	–
A. terreus	–	–	–	27.48
DW	–	–	–	–

Note: Ct, cycle threshold; "–" indicates no amplification detected within 40 PCR cycles; DW, distilled water (negative control).

Supplementary Table S2. Optimization of PCR cycling conditions for the MoiM *Aspergillus* 4-plex assay.

Condition	Initial Denaturation	Denaturation	Annealing /Extension	Detection results (Ct value)			
				A. fumigatus	*A. flavus*	*A. niger*	*A. terreus*
Condition 1	97℃/ 5 min	95℃/ 7 sec	63℃/ 20 sec	30.83	35.88	40.37	35.69
Condition 2	97℃/ 5 min	97℃/ 7 sec	63℃/ 20 sec	30.89	36.41	39.99	36.74
Condition 3	95℃/ 5 min	95℃/ 3 sec	63℃/ 20 sec	31.24	36.74	43.05	39.09
Condition 4	95℃/ 5 min	95℃/ 5 sec	63℃/ 20 sec	31.09	35.79	42.51	35.86
Condition 5	95℃/ 7 min	95℃/ 7 sec	63℃/ 20 sec	31.07	36.60	42.23	36.31
Condition 6	95℃/ 5 min	95℃/ 9 sec	63℃/ 20 sec	31.02	36.43	39.20	35.51
Condition 7	95℃/ 3 min	95℃/ 7 sec	63℃/ 20 sec	31.39	35.78	Not detected	36.41
Condition 8	95℃/ 1 min	95℃/ 7 sec	63℃/ 20 sec	34.20	38.16	Not detected	37.14

Note: Condition 6 was selected as the final protocol.

Supplementary Table S3. Analytical specificity of the *Aspergillus* 4-plex assay

	Strain	Concentration (copies/reaction)	Result
1	*Streptococcus mutans*	1x10[5]	Negative
2	*Escherichia coli*		Negative
3	*Staphylococcus aureus*		Negative
4	*Staphylococcus epidermidis*		Negative
5	*Staphylococcus warneri*		Negative
6	*Pseudomonas aeruginosa*		Negative
7	*Klebsiella pneumoniae*		Negative
8	*Alcaligenes faecalis* subsp. *faecalis*		Negative
9	*Proteus mirabilis*		Negative
10	*Bacillus cereus*		Negative
11	*Streptomyces griseoruber*		Negative
12	*Candida albicans*		Negative
13	*Candida auris*		Negative
14	*Candida dubliniensis*		Negative
15	*Candida glabrata*		Negative
16	*Candida guilliermondii*		Negative
17	*Candida krusei*		Negative
18	*Candida parapsilosis*		Negative
19	*Candida tropicalis*		Negative
20	*Candida lusitaniae*		Negative

Supplementary Table S4. Details of 22 bronchoalveolar lavage samples collected from invasive pulmonary aspergillosis (IPA) patients

Sample	Galactomannan test (O.D. results)	Anti fungal treatment	Category	Culture Results	MoiM Aspergillus 4-plex
1	> 10.0	Voriconazole	Proven IPA	*A. fumigatus*	*A. fumigatus*
2	> 10.0	Voriconazole	Proven IPA	*A. fumigatus*	*A. fumigatus*
3	> 10.0	Voriconazole	Proven IPA	*A. fumigatus*	*A. fumigatus*
4	> 10.0	Voriconazole	Proven IPA	*A. fumigatus*	*A. fumigatus*
5	> 10.0	Voriconazole	Proven IPA	*A. fumigatus*	*A. fumigatus*
6	> 10.0	Voriconazole	Proven IPA	*A. fumigatus*	*A. fumigatus/A. flavus*
7	> 10.0	voriconazole	Proven IPA	*A. fumigatus*	Negative
8	> 10.0	Micafungin, voriconazole, Amphotericin B	Proven IPA	*A. fumigatus*	*A. fumigatus*
9	> 10.0	Micafungin, voriconazole, Amphotericin B	Proven IPA	*A. fumigatus*	*A. fumigatus/A. terreus*
10	> 10.0	Itraconazole, voriconazole	Proven IPA	*A. fumigatus*	*A. fumigatus*
11	> 10.0	Itraconazole, voriconazole	Proven IPA	*A. niger*	*A. niger*
12	> 10.0	Voriconazole	Probable IPA	No growth	-
13	> 10.0	Voriconazole	Probable IPA	No growth	*A. fumigatus*
14	> 10.0	Voriconazole	Probable IPA	No growth	-
15	> 10.0	Voriconazole	Probable IPA	No growth	-
16	3.40	Voriconazole	Probable IPA	No growth	-
17	1.76	Voriconazole	Probable IPA	No growth	*A. flavus*
18	1.29	Voriconazole	Probable IPA	No growth	-
19	1.06	Voriconazole	Probable IPA	No growth	-
20	1.01	Amphotericin B	Probable IPA	No growth	-
21	0.69	Voriconazole	Probable IPA	No growth	*A. fumigatus*
22	0.63	Voriconazole	Probable IPA	No growth	-

Supplementary Table S5. Results of MoiM *Aspergillus* 4-plex assay using additional 67 BAL samples obtained from patients without antifungal therapy or clinical signs of IPA

Sample #	Galactomannan test (O.D. results)	Culture Results	MoiM Aspergillus 4-plex
1	> 10.0	Yeast, not *Cryptococcus*	-
2	> 10.0	Yeast, not *Cryptococcus*	-
3	> 10.0	No growth	-
4	> 10.0	Yeast, not *Cryptococcus*	-
5	> 10.0	Yeast, not *Cryptococcus*	-
6	> 10.0	No growth	-
7	> 10.0	No growth	-
8	> 10.0	No growth	-
9	> 10.0	Yeast, not *Cryptococcus*	-
10	> 10.0	Yeast, not *Cryptococcus*	-
11	> 10.0	No growth	-
12	> 10.0	No growth	-
13	> 10.0	No growth	-
14	3.37	No growth	-
15	2.20	Yeast, not *Cryptococcus*	-
16	1.99	No growth	-
17	1.71	No growth	-
18	1.67	No growth	-
19	1.66	No growth	-
20	1.51	No growth	-
21	1.46	No growth	-
22	1.46	Yeast, not *Cryptococcus*	-
23	1.41	No growth	*A. terreus*
24	1.37	Yeast, not *Cryptococcus*	-
25	1.33	No growth	-
26	1.22	No growth	-
27	1.00	No growth	-
28	0.25	No growth	-
29	0.19	No growth	-
30	0.19	No growth	-
31	0.18	No growth	-
32	0.17	Yeast, not *Cryptococcus*	-
33	0.17	No growth	-
34	0.16	No growth	-

35	0.16	No growth	-
36	0.15	Yeast, not *Cryptococcus*, *Penicillium commune*	-
37	0.15	No growth	*A. flavus*
38	0.15	No growth	-
39	0.15	Yeast, not *Cryptococcus*	-
40	0.15	No growth	-
41	0.14	No growth	-
42	0.14	No growth	-
43	0.13	No growth	-
44	0.13	No growth	-
45	0.13	No growth	*A. flavus*
46	0.13	No growth	-
47	0.12	No growth	-
48	0.12	No growth	-
49	0.11	No growth	-
50	0.11	Yeast, not *Cryptococcus*	-
51	0.11	No growth	-
52	0.11	No growth	-
53	0.11	No growth	-
54	0.10	Yeast, not *Cryptococcus*	-
55	0.10	No growth	-
56	0.10	No growth	-
57	0.08	No growth	-
58	0.07	No growth	-
59	0.06	Yeast, not *Cryptococcus*	-
60	0.06	Yeast, not *Cryptococcus*	-
61	0.05	Yeast, not *Cryptococcus*	-
62	0.05	No growth	-
63	0.05	No growth	-
64	0.05	No growth	-
65	0.04	Yeast, not *Cryptococcus*	-
66	0.03	No growth	-
67	0.03	Yeast, not *Cryptococcus*	-
